# Late Bilinguals Are Sensitive to Unique Aspects of Second Language Processing: Evidence from Clitic Pronouns Word-Order

**DOI:** 10.3389/fpsyg.2017.00342

**Published:** 2017-03-17

**Authors:** Eleonora Rossi, Michele Diaz, Judith F. Kroll, Paola E. Dussias

**Affiliations:** ^1^Department of Psychology and Sociology, California State Polytechnic University, Pomona, CAUSA; ^2^Department of Psychology, Pennsylvania State University, State College, PAUSA; ^3^Department of Psychology, University of California, Riverside, Riverside, CAUSA; ^4^Department of Spanish, Italian, and Portuguese, Pennsylvania State University, State College, PAUSA

**Keywords:** bilingualism, sentence processing, second language acquisition, clitic pronouns, L2 attainment

## Abstract

In two self-paced reading experiments we asked whether late, highly proficient, English–Spanish bilinguals are able to process language-specific morpho-syntactic information in their second language (L2). The processing of Spanish clitic pronouns’ word order was tested in two sentential constructions. Experiment 1 showed that English–Spanish bilinguals performed similarly to Spanish–English bilinguals and revealed sensitivity to word order violations for a grammatical structure unique to the L2. Experiment 2 replicated the pattern observed for native speakers in Experiment 1 with a group of monolingual Spanish speakers, demonstrating the stability of processing clitic pronouns in the native language. Taken together, the results show that late bilinguals can process aspects of grammar that are encoded in L2-specific linguistic constructions even when the structure is relatively subtle and not affected for native speakers by the presence of a second language.

## Late Bilinguals Are Sensitive to Subtle Aspects of Second Language Morphosyntax

A longstanding question about language learning is whether adults who acquire a second language (L2) after the hypothesized sensitive period for language acquisition are able to access grammatical structures in a native-like manner (e.g., Long, 1990; Birdsong, 1999). The evidence from past studies using sentence judgment tasks is mixed, showing that speakers who learn an L2 later in life are often less sensitive than native speakers in identifying grammatical violations (e.g., Johnson and Newport, 1991). Some have argued that late L2 speakers differ from native speakers in how they process grammatical structures that are unique to the L2 and not readily transferable from the L1 (e.g., Johnson and Newport, 1991; Weber-Fox and Neville, 1996; MacWhinney, 2005; Clahsen and Felser, 2006; Sabourin et al., 2006; Sabourin and Stowe, 2008). On this account, native-like processing of L2 grammar is hypothesized to be difficult to achieve (Herschensohn, 2001; Clahsen and Felser, 2006). An alternative proposes that native-like processing is possible, especially when L2 speakers achieve a high level of proficiency (e.g., McDonald, 2000; Birdsong and Molis, 2001; Coughlin and Tremblay, 2013), or when L2 speakers are matched in working memory capacities to native speakers (Hopp, 2013).

A problem in adjudicating the debate about the ability of late bilinguals to access L2 grammar is that much of the past research has relied on off-line behavioral measures of sentence processing that represent an aggregate of linguistic and cognitive factors, making it difficult to disentangle the mechanisms that guide real-time processing. When studies have employed temporally sensitive measures (self-paced reading, eye tracking, and Event related Potentials, ERPs), L2 learners have demonstrated native-like processing of subtle aspects of the L2 grammar (e.g., Birdsong and Molis, 2001; Hopp, 2010; McLaughlin et al., 2010; Morgan-Short et al., 2010; Coughlin and Tremblay, 2013; Rossi et al., 2014). However, the observation that highly proficient late bilinguals can achieve native-like performance does not necessarily mean that they rely on the same processing strategies as native speakers, especially for those structures that may impose language-specific constraints (e.g., Clahsen and Felser, 2006).

To examine this issue, we investigated the ability of proficient, but late, English–Spanish bilinguals in processing clitic pronouns, a grammatical structure that is present in Spanish, the L2, but not in English, the L1. Clitic pronouns have been shown to be a particularly taxing structure for native speakers acquiring their L1 (Pérez-Leroux et al., 2011), a vulnerable structure for agrammatic speakers (Rossi, 2013), and a complex grammatical structure to acquire for L2 learners whose L1 does not represent it (e.g., Grüter, 2006; Santoro, 2007; Grüter and Crago, 2012; Coughlin and Tremblay, 2013; Rossi et al., 2014).

Spanish clitic pronouns are marked for grammatical gender and number, features that have to match the antecedent (for example in Spanish: “Ana tome *la* manzana y se *la* puso en la bolsa”; Ana took the apple and put it in the bag). In the context of the present research, not only are clitics absent as a linguistic structure in English, but gender is also not marked in English, making it difficult to transfer from the L1 to the L2.

Research on L2 gender and number processing across a variety of grammatical constructions has yielded mixed results. Some studies suggest incomplete acquisition (e.g., Sabourin et al., 2006; Montrul et al., 2008), while others demonstrate that proficient L2 speakers with extensive immersion experience appear to process gender qualitatively similarly to native speakers (Dowens et al., 2011). For gender and number marked on clitic pronouns, Coughlin and Tremblay (2013) reported that both intermediate and highly proficient English L2 learners of French are sensitive to violations of number agreement when presented via an off-line grammaticality judgment task, while only highly proficient L2 learners show sensitivity to the number violations in the context of an on-line self-paced reading task. Similarly, Santoro (2007) found that English intermediate learners of Italian responded more accurately than beginning learners to violation of case marking and placement during an off-line grammaticality judgment task, suggesting that even late L2 learners can improve in the acquisition of L2 specific grammatical structures.

In a recent series of neurophysiological studies, Rossi et al. (2014) and Rossi and Prystauka (unpublished) investigated the real time processing of clitic pronouns using ERPs and the oscillatory frequency-based signal in both native Spanish speakers and in late English–Spanish bilinguals. The primary goal of those studies was to determine sensitivity to grammatical gender and number marked on clitic pronouns while participants processed sentences containing clitic pronouns which either correctly matched the antecedent in gender and number, or violated gender agreement or number agreement or both. ERP data revealed that proficient L2 speakers as a group were sensitive to violations of number but not grammatical gender. However, a subgroup of very-high proficiency L2 speakers showed similar sensitivity to violations of grammatical gender as native speakers (as indexed by a modulation of the P600 component), suggesting that at least a subset of late L2 bilinguals was able to achieve native-like processing for structures not present in their L1. The oscillatory results (Rossi and Prystauka, unpublished) confirmed the ERP data and demonstrated that L2 speakers were sensitive to both number and gender violations, as revealed by a decrease in power in the beta frequency band (11–22 Hz for number violations, and 14–21 Hz for gender violations), which has been reported in past research for grammatical violations (Davidson and Indefrey, 2007). Taken together, recent behavioral and neurophysiological evidence demonstrates that late but highly proficient bilinguals are sensitive to clitic pronouns, a feature of the L2 morphosyntax not present in the L1, and also to the grammatical features that are marked onto them, such as grammatical gender and number.

Rossi et al. (2014) and Rossi and Prystauka (unpublished) investigated late bilinguals’ sensitivity to number and gender marked on clitics in finite sentences. Critically, in those studies number and grammatical gender features were violated, but clitic position was not manipulated. Importantly, clitic pronouns in Spanish may appear in different positions depending on the sentential construction. They appear obligatorily before finite verbs in finite sentence, as in: *Ana lo come* (Anna eats it), a word order that does not map onto the word order for English weak pronouns, which appear post-verbally in finite sentences (i.e., *Mary eats it*). In other sentential constructions, however, the surface order of Spanish clitics and English weak pronouns overlap, for example with an infinitival verb preceded by a modal, causative or aspectual verb (traditionally referred as ‘restructuring verbal constructions’; Rizzi, 1982). Previous behavioral research on the L2 acquisition of Spanish clitic placement has shown that intermediate L2 speakers of Spanish are relatively successful in acquiring L2 clitic placement, when measured by off-line grammaticality judgment tasks (Duffield and White, 1999; Duffield et al., 2002; Montrul, 2010), sentence matching tasks (Duffield and White, 1999; Duffield et al., 2002), and speeded visual-picture matching task (Montrul, 2010). However, those studies did not examine the on-line course of clitic placement processing.

The primary goal of the present study is to examine sensitivity to word order for clitic pronouns in late English–Spanish bilinguals using a non-cumulative word-by-word self-paced reading paradigm to assess linguistic performance in real-time (White and Juffs, 1998; Leeser et al., 2011; Jegerski, 2014). Sensitivity to Spanish clitic word order represents a strong test of L2 acquisition because of the uniqueness of this grammatical structure and the partial difference in surfacing word order between Spanish clitics and English weak pronouns. Here, we report two experiments. In Experiment 1, we ask whether late English–Spanish bilinguals are sensitive to clitic pronouns and their surface word order. We compare their performance to a group of native Spanish speaking bilinguals living in the US, immersed in English as the L2. Because recent studies have suggested that the frequency of use in response to L2 exposure and language immersion can influence L1 lexical processing and parsing preferences (e.g., Dussias, 2003; Malt and Sloman, 2003; Dussias and Sagarra, 2007; Schmid, 2010; Rossi and Diaz, 2016; Rossi et al., unpublished) it was important to determine whether the sensitivity to the clitic structure and its word order remained unchanged for native speakers of Spanish across different language contexts. In Experiment 2, we therefore replicated the design of Experiment 1 with functionally monolingual speakers of Spanish living in Spain. The primary goal of Experiment 2 was to assess the stability of clitic processing in Spanish under different conditions of language use. Experiment 2 also enabled us to compare the late bilinguals in Experiment 1 with a group of native Spanish speakers who were more closely matched in age. In what follows we first provide an introduction to clitic pronouns, report the two experiments, and then discuss the results in relation to the current literature on L2 processing.

### The Spanish Clitic Pronoun System

Spanish clitics are pronominal particles that stand in place of the full noun. Spanish clitics encode grammatical gender (“lo,” *it Masculine Singular*; “la,” *it Feminine Singular*) and number (“los,” *it Masculine Plural*; “las,” *it Feminine Plural*). Additionally, Spanish clitics appear in different sentential position. They obligatorily appear before finite verbs in finite sentential constructions [as in (1)], and after the verb in restructuring constructions (Cardinaletti and Shlonsky, 2004), which are sentential constructions, formed by an infinitival verb preceded by a modal, causative or an aspectual verb [as in (2)]. It is important to note that restructuring sentences allow also for the clitic pronoun to be placed before the modal (causative or aspectual) verb, as for example in “Antes de comer la pizza, Teresa *la* quiso calentar en el horno” (clitic climbing). However, in the present study, we will focus on post-infinitival constructions (see Rossi, 2013 for a priming study on clitic climbing with agrammatic speakers).

#### Finite constructions

(1) Antes de comer el mango, Juan lo peló con un cuchillo
     *Before eating the mango it (Masculine Singular Clitic), Juan it (Masculine Singular Clitic) peeled with a knife* “Before eating the mango, Juan peeled it with a knife”

Modal, causative, aspectual + infinitival (Restructuring constructions)

(2) Antes de comer la pizza, Teresa quiso calentarla en el horno
     *Before eating the pizza (Feminine Singular Clitic), Teresa wanted to heat it (Feminine Singular Clitic) in the oven*
     “Before eating the pizza, Teresa wanted to heat it in the oven”

In the experiments we report, participants read finite and restructuring constructions in Spanish in which direct object clitic pronouns (varying in both gender and number) were presented either in the correct or incorrect positions in the sentence. For finite constructions, word order was violated by placing direct object clitics after the finite verb [as in (3b)], while it appeared before the finite verb in the correct condition (3a)

(3) (a) Antes de leer el libro, Ana lo sacó de la envoltura de plástico
      (b) * Antes de leer el libro, Ana sacó lo de la envoltura de plástico
      *Before reading the book, Ana took it out of the plastic cover*

For restructuring constructions (in the correct condition) we chose to be consistent and use a post-verbal clitic position, in which the clitic appears merged with the verb (4a). To create the violation for the incorrect condition, the clitic was placed between the finite and the non-finite verb (4b).

(4) (a) Después de haber comprado los mangos, Ana decidió guardarlos en la nevera
      (b) * Después de haber comprado los mangos, Ana decidió los guardar en la nevera
      *After having bought the mangos, Ana decided to keep them in the fridge*

Violations of clitic position in these two types of constructions allowed us to test predictions regarding sensitivity to clitic pronouns for both L1 and L2 speakers, and also to test predictions regarding transfer between the L1 and the L2.

## Experiment 1: Clitic Processing in Spanish as an L1 or L2

Experiment 1 compared sensitivity to Spanish clitic pronouns for proficient but late English–Spanish bilinguals and native Spanish-speaking bilinguals immersed in an English speaking environment in the US. Each group read finite and restructuring sentences in Spanish in which the position of direct-object clitics was either correct or incorrect. Because Spanish clitic pronouns encode morphosyntactic features that are not present in English (i.e., grammatical gender) and sometimes require a word order that does not map onto English weak pronouns (i.e., in finite sentential constructions), they provide an ideal test of the hypothesis that late bilinguals may be constrained in their sensitivity to the grammar of the L2.

If late L2 speakers are sensitive to the clitic pronoun and to their surfacing word order, we predict that they will show longer reading times for the experimental conditions at the point at which the clitic position will be violated. Conversely, if late L2 speakers are not sensitive to clitics and their surfacing word order, we would predict no difference in reading times between the correct and the incorrect conditions. We expect that native Spanish speakers should be sensitive to violations of clitic word order for both the finite and the restructuring condition.

### Method

#### Participants

The study was approved by the IRB board of Penn State University. Twenty-five native speakers of Spanish proficient in English as the L2 (14 females, 11 males; mean age: 29.5 years.; age range: 19–43; *SD* = 8.6) immersed in the L2 in the US (mean length immersion: 4 years) and 25 late English–Spanish bilinguals (19 females, 6 males; mean age: 23.5 years; age range: 19–34; *SD* = 3.7; mean age of acquisition: 14.7; age range: 12–19; *SD* = 3.5) were recruited. Both groups were students at the Pennsylvania State University and were paid for their participation. None reported any neurological or reading disorder and had normal or corrected-to-normal visual acuity. Participants completed a language history questionnaire to assess their language history and skills in both languages. They rated their language knowledge using a scale from 1 to 10 (1 being the lowest and 10 being the highest score) for oral comprehension, oral production, reading and writing. Additionally, the grammar section of the Diploma de Español como Lengua Extranjera (DELE, Ministry of Education Culture and Sport of Spain, 2006) was also administered to obtain an objective measure of grammatical knowledge in Spanish.

From the initial pool of participants, three heritage speakers (who were born in the US and grew up in a bilingual environment) and three native Spanish speakers who had become dominant in English (self-reporting higher or equal proficiency in English than Spanish) were excluded. Consequently, 19 native Spanish speakers were included in the final analyses. For the English–Spanish bilinguals, participants who self-rated their Spanish abilities on average below seven, who scored below 35% in the DELE test and who scored below 85% on the experimental task were also excluded from the final analyses. In all, five participants were excluded, resulting in a total of 20 proficient English–Spanish bilinguals. The characteristics of the two bilingual groups are summarized in **Table 1**.

**Table 1:** Mean values for the self-rating scores and the DELE scores for all groups.

	Experiment 1: Spanish–English bilinguals Mean (*SD*)	Experiment 1: English–Spanish bilinguals Mean (*SD*)	Experiment 2: Monolingual Spanish Mean (*SD*)
**English**
Oral production (0 min–10 max)	8.3 (1.2)	9.9 (0.2)	4.2 (1.4)
Oral comprehension (0 min–10 max)	8.6 (0.9)	9.9 (0.2)	3.7 (1.6)
Reading (0 min–10 max)	8.7 (0.8)	9.9 (0.2)	5.0 (1.2)
Writing (0 min–10 max)	8.0 (0.9)	9.9 (0.4)	4.9 (1.5)
Mean English Rating	8.4 (0.7)	9.9 (0.2)	4.5 (1.3)
**Spanish**
Oral production (0 min–10 max)	9.9 (0.3)	7.3 (1.1)	9.3 (0.9)
Oral comprehension (0 min–10 max)	10.0 (0)	7.8 (1.1)	9.6 (0.6)
Reading (0 min–10 max)	9.8 (0.5)	7.7 (1.1)	9.3 (0.9)
Writing (0 min–10 max)	9.7 (0.7)	7.2 (1.2)	9.3 (0.8)
Mean Spanish Rating	9.9 (0.3)	7.5 (1)	9.4 (0.7)
DELE	0.8 (0.1)	0.6 (0.1)	0.9 (0.05)

#### Materials and Design

One hundred and sixty experimental sentences were created (80 experimental stimuli and 80 fillers). Examples of the stimuli are presented in **Table 2**. Half of the critical experimental items (*n* = 40) were finite sentences and the other half (*n* = 40) were restructuring sentences. For both sentence types, clitic pronouns were presented in the correct sentential position (*n* = 20) or in the incorrect position (*n* = 20). Other than the experimental manipulation, the general structure of the sentences was held constant. Sentences began with a preamble, containing an antecedent (a determiner and a noun), followed by a clitic pronoun which appeared in either the correct or in an incorrect position. The clitic pronoun always matched the antecedent in gender and number. As mentioned, in finite sentences the position of clitic pronouns was violated by placing the clitic after the finite verb. For restructuring sentences, the position was violated by placing the clitic before the non-finite verb. Experimental sentences varied from 10 to 14 words. All the critical portions in the experimental sentences (i.e., the noun preceding the clitic and the verb, the verbs, and the preposition following those) were matched for word length and word frequency, to ensure equality of stimuli across conditions. Specifically, word frequency (log) and word length (in letters) were checked for four words preceding and for two words following the critical word (the clitic) using word values available in the NIM database (Guasch et al., 2013) to ensure homogeneity across experimental lists. There were no statistically significant differences in frequency or length for any of these words categories, besides the length of the proper nouns (such as Ana, Carlos) which was, however, more difficult to control because the material was chosen with the goal of having different proper names in each experimental item, to make the materials more varied. Also, the word frequency for proper nouns was not analyzed, because of the relative difficulty to establish frequency for proper names (word length first noun: *F* = 0.54; *p*s = 0.65 n.s.; word length proper noun: *F* = 7.01; *p*s < 0.05; length in letters for verb after the clitic: *F* = 0.95; *p*s = 0.41 n.s; word frequency first noun: *F* = 2.9; *p*s = 0.048; frequency verb after the clitic in letters: *F* = 1.26; *p*s = 0.29 n.s). Gender (feminine and masculine) and number (singular and plural) at the clitic were also counterbalanced equally across experimental items. Finally, to avoid end-of sentence processing effects, at least three words followed the critical region of interest. Gender (feminine and masculine) and number (singular and plural) were counterbalanced across experimental items.

**Table 2:** Examples of the experimental sentences for finite and restructuring constructions.

	Correct clitic position	Incorrect clitic position
**Finite constructions**
Masculine singular clitic	Antes de leer el libro, Ana lo sacó de la envoltura de plástico	Antes de leer el libro, Ana sacó lo de la envoltura de plástico
Masculine plural clitic	Antes de leer los libros, Ana los sacó de la envoltura de plástico	Antes de leer los libros, Ana sacó los de la envoltura de plástico
Feminine singular clitic	Antes de comer la manzana, Ana la peló con un cuchillo	Antes de comer la manzana, Ana peló la con un cuchillo
Feminine plural clitic	Antes de comer las manzanas, Ana las peló con un cuchillo	Antes de comer las manzanas, Ana peló las con un cuchillo
**Restructuring constructions**
Masculine singular clitic	Después de haber comprado el mango, Ana decidió guardarlo en la nevera	Después de haber comprado el mango, Ana decidió lo guardar en la nevera
Masculine plural clitic	Después de haber comprado los mangos, Ana decidió guardarlos en la nevera	Después de haber comprado los mangos, Ana decidió los guardar en la nevera
Feminine singular clitic	Antes de comer la pizza, Teresa quiso calentarla en el horno	Antes de comer la pizza, Teresa quiso la calentar en el horno
Feminine plural clitic	Antes de comer las pizzas, Teresa quiso calentarlas en el horno	Antes de comer las pizzas, Teresa quiso las calentar en el horno

Eighty filler sentences were included (40 grammatical and 40 ungrammatical) in an attempt to prevent participants from noticing the manipulation of interest. Fillers included a range of structures such as subject-verb agreement, mood agreement, and sentences with high and low attachment preferences. There were 20 comprehension questions that were created to ensure that participants were performing the reading task appropriately. Comprehension questions appeared following 9% of the trials, and were distributed at regular intervals across conditions. Unbeknownst to the participants, the comprehension questions referred only to filler sentences. For the remaining trials, participants saw the following sentence: “*No hay pregunta. Continúa”; “There is no question. Continue”* which prompted them to continue to the next sentence.

There were two experimental lists, with the same sentences correct in one list and incorrect in the other. A given participant saw only one of the two versions of the sentences. The presentation of sentences was pseudo-randomized so that consecutive trials were not: (a) two experimental sentences; (b) two finite or two restructuring sentences; or (c) two sentences containing a feminine or a masculine (singular or plural) clitic. Finally, 12 practice items (six correct and six incorrect) preceded the task to familiarize participants with the task.

#### Procedure

Sentences were presented using a non-cumulative word-by-word self-paced reading moving window task (Just et al., 1982). Participants were instructed to read and to perform a grammaticality judgment at the end of each sentence as fast and as accurately as possible. The prompt to perform the grammaticality judgment was presented in a separate frame, after the end of the sentence. Participants were also told to read each sentence carefully, as they would be asked to answer periodic comprehension questions. Each trial started with a fixation cross in the middle of the screen. Once the space bar was pressed, the first word appeared. The remaining words in the sentence were represented by dashes (one dash representing each letter of each word). Words were visually separated by a space. They proceeded through the sentence by pressing the space bar, one word at a time. Once they went on to the next word, the preceding word disappeared. The time that elapsed between the onset a word and each subsequent word was recorded. Each sentence was followed by the question: “¿La oración es gramatical?” (Is the sentence grammatical?) which appeared on a separate screen. Participants were asked to indicate whether the sentence was grammatical as quickly and accurately as possible by pressing “yes” or “no” response keys. Response Times (RTs) and accuracy for the grammaticality judgments were collected. For a subset of filler sentences, comprehension questions followed the grammaticality judgments.

The decision to use a non-cumulative word-by-word self-paced reading paradigm was motivated by previous studies which used this technique to measure linguistic performance to complement grammaticality judgments as a measure of linguistic competence (White and Juffs, 1998; Leeser et al., 2011; Jegerski, 2014). The goal was to measure reading times at the critical regions of interest (ROI) reflecting on-line implicit grammatical processing, and also to collect an additional measure of explicit linguistic performance. To parallel the design in Rossi et al. (2014) we used an end of the sentence acceptability judgment task. Importantly, Rossi et al. (2014) showed differential sensitivity to the clitic structure in native Spanish speaker and in English–Spanish bilinguals as revealed by the presence of a P600 component, reflecting real-time sensitivity to the clitic structure, even with an off-line acceptability task. ERPs are a direct reflection of on-line processing, and they reflect implicit brain responses to stimuli (Osterhout et al., 1997).

#### Statistical Analysis

A similar set of statistical analyses was performed for both experiments. The first step was to examine the accuracy and speed of the end of the sentence grammaticality judgments. A series of 2 × 2 repeated measures ANOVAs with Correctness [correct or incorrect clitic position], and Speaker Group [L1 or L2 for Experiment 1; L1 and Monolinguals for Experiment 2] as factors were performed on only correct trials only for finite and restructuring sentences, after the exclusion of absolute and relative outliers (absolute outliers:<300 ms or >5000 ms; relative outliers: ± 2.5 SD from each participant’s mean). To simplify the report of the results within the text, only significant results will be reported by subjects (F1) and by items (F2). Additionally, self-paced reading data were examined at three selected ROIs as a measure of on-line processing. For finite sentences, the first ROI was defined by the clitic-verb compound (for correct items), or as the verb-clitic compound (for incorrect items). Despite the fact that the clitic and the verb were presented as separate words during the task, compounding them for analysis purposes allowed us to eliminate any baseline problems that would have arisen by analyzing them separately. The second ROI was identified as the word immediately following the first ROI, and the third as the second word following the first ROI. For example, for the finite sentence: “Antes de leer el libro, Ana lo sacó de la envoltura de plástico” (before reading the book, Ana took it out of the plastic cover) the first ROI was “lo sacó” or “sacó lo,” in the correct and incorrect conditions respectively. The second ROI was “de” and the third ROI “la.” For restructuring sentences, the first ROI was defined as the verb-clitic compound site, the second was the word following the first ROI (verb-clitic), and the third was the second word following the first ROI. For example, for the item: “Después de haber comprado los mangos, Ana decidió guardarlos en la nevera (after having bought the mangos, Ana decided to keep them in the fridge), the first ROI was “guardarlos” or “los guardar,” in the correct and incorrect conditions respectively. The second ROI was “en” and the third ROI “la.” It is important to note that the baseline of the two structures (e.g., the linguistic structure before the critical clitic pronoun) are very different, with the clitic in the finite construction being preceded by a proper noun, and the clitic in the restructuring sentence being preceded by a modal verb and the main verb. As such, we reasoned that a direct comparison between the two structures would not have been licit. The same baseline issues in comparing finite and restructuring constructions arise in the manipulated incorrect condition. As such we will perform two separate analyses, one for each sentential structure.

### Results

#### End-Sentence Grammaticality Judgments

##### Accuracy

L1 Spanish speakers were equally accurate than L2 Spanish speakers in detecting the violation of clitic position in finite sentences [finite sentences: L1 Spanish: 98.4%; L2 Spanish speakers: 93.1%; *F*(1,37) = 0.2; *p* = n.s]. The analysis revealed also a significant main effect of Group [*F*(1,37) = 13.229; *p* = 0.003], with native speakers being overall more accurate than English-Spanish bilinguals, but there was no significant Group by Correctness interaction. For restructuring sentences despite the apparent difference in performance (L1 Spanish speakers: 97.2%; L2 Spanish speakers: 94.5%) the analysis did not show any differences in performance between the two groups [*F*(1,37) = 2.7; *p* = n.s].

##### Response times

For finite sentences there were no significant effects. Grammaticality judgments were similar for correct and incorrect sentences [*F*1(1,37) = 0.532, MSE = 43,489; *p* = 0.470; *F*2(1,9) = 1.756, MSE = 11,929; *p* = 0.218]. There were no interactions with Group.

For restructuring sentences, there were no significant effects, and no interactions by Group. End of the sentence grammaticality judgments’ RTs were similar for correct and incorrect sentences [*F*1(1,37) = 0.217, MSE = 40,847; *p* = 0.644; *F*2(1,9) = 3.105, MSE = 26,566; *p* = 0.112]. There were no interactions with group.

Based on grammaticality judgment performance alone, we might argue that L2 speakers resemble native speakers. Although the L2 speakers were slightly less accurate than the native speakers, their scores were above 90% for accuracy, indicating sensitivity to the two types of violations. However, RTs measured in the context of grammaticality judgments likely reflect an aggregate of effects that may mask critical group differences. The self-paced reading data, with RTs recorded at different regions of the sentence, provide a more sensitive measure of on-line processing, and thus may be more likely to reveal fine-grain similarities and differences across the two groups.

#### Self-paced Reading Data

**FIGURE 1:**
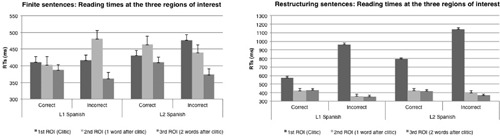
**Experiment 1: Reading times at the three regions of interest for finite and restructuring constructions in native Spanish speakers and English–Spanish bilinguals**.

In what follows we report the results for finite constructions first, followed by those for restructuring constructions. We will report the most relevant results. A complete table with all the results with F1, F2, MSE values and post-hoc test statistics is provided in Appendix A. Results for both constructions are given in **Figure 1**.

##### Finite constructions

At the first ROI (i.e., clitic + verb in the correct condition or verb + clitic in the incorrect condition) a main effect of Correctness revealed that participants’ reading times were significantly slower in the incorrect condition (i.e., when the clitic was presented after the verb) [*F*1(1,37) = 11.654; *p* = 0.002; *F*2(1,39) = 13.371; *p* = 0.001]. A significant Correctness by Group interaction in the analysis by subject, followed by pairwise *post hoc* analyses suggest that only English–Spanish bilinguals were significantly slower when reading clitics in the incorrect position in their L2, [*F*1(1,37) = 7.148; *p* = 0.001]. The analysis by item did reveal a main effect of Group [*F*2(1,39) = 13.422; *p* = 0.001], showing overall that L2 speakers were slower, but the interaction with Correctness did not reach significance *F*2(1,39) = 2.518; *p* = n.s.). At the second ROI (i.e., the word following the first ROI composed by the clitic + verb) a significant Correctness by Group interaction [*F*1(1,37) = 7.22; *p* = 0.011; *F*2(1,39) = 20.21; *p* = 0.001] followed by pairwise *post hoc* analyses revealed that the L1 group showed longer reading times for sentences in which the clitic was presented in an incorrect condition, while L2 bilinguals did not show such effect. Finally, at the third ROI (i.e., the second word following the first ROI) a main effect of Correctness in the analysis by subject and a trending significance in the analysis by item [*F*1(1,37) = 8.795; *p* = 0.005; *F*2(1,39) = 3.51; *p* = 0.068] showed that words following the incorrect condition were read faster than words following the correct condition. There was no significant Correctness by Group interaction.

##### Restructuring constructions

At the first ROI (i.e., clitic + verb in the correct condition or verb + clitic in the incorrect condition) a main effect of Correctness showed that clitics in the incorrect position were read slower than clitics in the correct position [*F*1(1,37) = 91.115; *p* < 0.01; *F*2(1,39) = 69.509; *p* < 0.01]. Moreover, the analysis by item revealed a significant Correctness by Group interaction [*F*2(1,39) = 5.32; *p* = 0.02], which was not significant at the subject level. At the second and third ROIs (i.e., the second and third words following the first ROI), there was a main effect of Correctness, with longer reading times for words following the correct condition relative to the incorrect condition [second ROI: *F*1(1,37) = 22.862; *p* < 0.01; *F*2(1,39) = 15; *p* < 0.01; third ROI: *F*1(1,37) = 36.954; *p* < 0.01; *F*2(1,39) = 7.964; *p* < 0.01]. Additionally, the analysis by item only showed a significant Correctness by Group interaction in the second ROI [*F*2(1,39) = 17.878; *p* < 0.01], showing longer reading times for incorrect experimental items in native speakers.

### Discussion

The data from Experiment 1 show that L1 and L2 speakers were overall sensitive to the violation of clitic position both in finite and restructuring constructions. Notably, because clitic pronouns are a grammatical structure that is unique to Spanish, the L2 for the late English–Spanish bilinguals in this study, the findings support the view that adult L2 learners who are sufficiently proficient show sensitivity to grammatical structures that are not present in their native L1. The present results are in line with previous off-line behavioral results that reported sensitivity to violations of clitic placement in late L2 speakers (Duffield and White, 1999; Duffield et al., 2002; Montrul, 2010). More broadly, they also corroborate recent behavioral and neurophysiological findings showing that relatively proficient L2 speakers are sensitive to L2-unique grammatical constructions (e.g., Santoro, 2007; Coughlin and Tremblay, 2013). Importantly, these results extend the results of Rossi et al. (2014) and Rossi and Prystauka (unpublished) on sensitivity to gender and number features marked on clitic pronouns, but which did not test sensitivity to clitic word order. Overall, the current results reveal L2 sensitivity to clitic placement, and support the view that native like attainment of L2 grammatical structures is possible, even when those structures are unique to the L2, and even when they are learned past early childhood.

Despite the overall similarity between native and the L2 speakers’ performance, the data reveal an important difference between the two groups. English–Spanish bilinguals show longer RTs at the first ROI for finite sentences (i.e., at the clitic), and a correctness by group interaction (significant at the subject level) highlights that English–Spanish bilinguals are slower when reading a clitic in the incorrect position. Importantly, at the second ROI we observed longer reading times for the L1 group for sentences in which the clitic was presented in an incorrect condition, while L2 bilinguals did not show such effect. A possible interpretation for the presence of the effect for L1 Spanish speakers only at the second ROI is that this might represent a delayed effect from the processing at the first ROI. An alternative, but related interpretation could be cast within a framework of maintenance and prediction. Let us spell out this proposal. In Spanish finite sentences, a clitic pronoun placed in a post-verbal position could potentially be interpreted as the determiner of a noun phrase, particularly if overlapping in form with the determiner counterpart (i.e., *la* feminine singular determiner; *la* feminine singular clitic, *las* feminine plural determiner; *las* feminine plural clitic; *los* masculine plural determiner; *los* masculine plural clitic) as in the following example: “Antes de pelar los mangos, Ana sacó los frutos de la envoltura de plastic” (Before peeling the mangos, Ana took the fruits out of the plastic bag). This is the case for all clitics except for the singular masculine clitic form “lo” which is different from its determiner counterpart “el” (i.e., *el* masculine singular determiner; *lo* masculine singular clitic). It could therefore be hypothesized that if readers temporarily interpret the post-verbal clitic as a potential determiner of an upcoming new noun (for example: “Antes de comer la manzana, Ana sacó *la* … banana…), they could show no effect at first at the clitic, but should show a spill-over effect at the word after the clitic, when they do not encounter the noun. Potentially, the same spill-over effect could be predicted for singular masculine clitic following a finite verb which could also be temporarily interpreted as correct (up to the clitic) if the misplaced clitic were to be processed as a relative clause, as in “Antes de leer el libro, Ana sacó *lo* que quería de su mochila” (Before reading the book, Ana took what she wanted from her backpack).

Although the primary goal of this study was not to test predictions regarding the use morphosyntactic information to predict upcoming information, we hypothesize that if the parser keeps these possibilities open, the temporary interpretation should fail (signaled by longer RTs) once a preposition is encountered after the first ROI rather than a noun following the post-verbal clitic. The results are in line with this suggestion because only native Spanish speakers showed longer reading times at the second ROI when the clitic position was previously violated, suggesting that only native speakers were able to maintain temporarily active alternative interpretations.

The results observed in processing restructuring sentences substantiate this proposal. The type of word order manipulation that was utilized (i.e., placing clitics that referred to a previously introduced noun phrase between the finite and the non-finite verb as in “*Después de haber comprado los mangos, Ana decidió los guardar en la nevera”) rarely allowed for an alternative interpretation. This explanation is supported by the results at the second ROI, where performance was mostly comparable between the two groups, even though the analysis by item at the second ROI revealed that L1 speakers had longer reading times for incorrect experimental items, which could be interpreted as a spill over effect from the first ROI. Overall, we suggest that a violation of clitic position in restructuring sentences does not allow for alternative interpretations to be maintained.

## Experiment 2: Sentence Processing in L1: The Effects of the Context of Language Use

In Experiment 1, we compared the performance of late English–Spanish bilinguals with native speakers of Spanish who were Spanish–English bilinguals immersed an L2 environment in the US. Although steps were taken to eliminate native Spanish speakers who were heritage speakers or who had become dominant in English as the L2, it is still possible that the picture of native language performance in Experiment 1 may have been affected by the fact of the native speakers’ active bilingualism while immersed in the L2.

A recent body of literature has demonstrated that the frequency of use and language immersion in the L2 can rapidly influence the availability of lexical information, and L1 parsing preferences in the L1 (e.g., Dussias, 2003; Malt and Sloman, 2003; Dussias and Sagarra, 2007; Linck et al., 2009; Schmid, 2010) suggesting that native language processing is highly malleable, especially in the context of L2 immersion, and that L1 processing mechanisms change and adapt depending on differential cognitive and language requirements (Green and Abutalebi, 2013; Kroll et al., 2015a). Moreover, the process of continuous adaptation that the native language undergoes as a result of bilingualism, and prolonged immersion in the L2, has been proposed to reflect one of the earliest adaptive stages of more radical and long-lasting changes in L1 processing, possibly leading to L1 attrition (e.g., Sorace and Filiaci, 2006; Keijzer, 2007; Sorace and Serratrice, 2009; Keijzer, 2010; Schmid, 2010; Rossi and Diaz, 2016; Rossi, et al., unpublished).

The goal of Experiment 2 was therefore to determine whether the pattern obtained for native speakers in Experiment 1 could be replicated with functionally monolingual speakers of Spanish living in Spain. Comparing performance between these two groups of speakers is of interest for two reasons. First, it will determine whether the clitic structure is open to change when the nature of language exposure and usage changes during L2 immersion. Although the similarity of the L1 and L2 readers in Experiment 1 suggests that both groups are sensitive to the violations of clitic placement, it is possible that some aspects of their performance were influenced by the English-dominant context. For example, it is possible that high frequency exposure to the English post-verbal pronouns, could result in diminishing the sensitivity to the incorrect clitic placement condition, making the groups appear similar. The comparison with native speakers of Spanish in their L1 environment will provide data on the stability of the clitic structure in the context of different language contexts. In addition, Experiment 2 enabled us to test a group of native Spanish speakers who were more closely matched in age to the native English-speaking late bilinguals in Experiment 1.

### Method

#### Participants

A group of 21 monolingual speakers of Spanish (14 females, 7 males; mean age: 21.6 years; age range: 18–36; *SD* = 4.67) were tested. Participants were recruited from the student population at the University of Granada (Spain) and given study credits for their participation. None reported any neurological or language disorder, and vision was normal or corrected-to-normal. Language history was assessed with the same questionnaire used in Experiment 1. One participant was excluded due to low accuracy on the grammaticality judgment task. Therefore, the data from a total of 20 monolingual Spanish speakers were analyzed. Performance of this new group of native speakers was compared to the Spanish–English bilinguals in Experiment 1 in **Table 1**.

#### Materials, Design, Procedure, and Data Analysis

Materials, design, procedure, and data analysis were the same as in Experiment 1.

### Results

#### End-Sentence Grammaticality Judgment Data

##### Accuracy

Spanish monolinguals were accurate on 97.3% of the grammaticality judgments (finite sentences: 97%; restructuring sentences 97.5%). The analysis did not reveal any differences in performance between the Spanish monolinguals tested in Spain and the Spanish–English bilinguals tested in the US (Finite sentences: Spanish monolinguals: 97%; Spanish-English bilinguals: 98.4% [*F*(1,37) = 2.17; *p* = n.s.]. Restructuring sentences: Spanish monolinguals: 97.5%; Spanish–English bilinguals: 97.2%; [*F*(1,37) = 0.08; *p* = n.s.].

##### Response times

For finite sentences there were no significant effects. Grammaticality judgments were similar for correct and incorrect sentences [*F*1(1,37) = 1.034, MSE = 13,252; *p* = 0.316; *F*2(1,9) = 0.418, MSE = 1,467; *p* = 0.0624]. There were no interactions with group. For restructuring sentences, there were also no significant effects, and no interactions with group. Grammaticality judgments were similar for correct and incorrect sentences [*F*1(1,37) = 0.084, MSE = 4,7881; *p* = 0.773; *F*2(1,9) = 1.205, MSE = 4,352; *p* = 0.0672]. There were no interactions with group.

#### Self-Paced Reading Data

**FIGURE 2:**
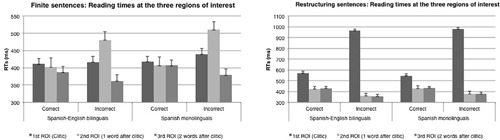
**Experiment 2: Reading times at the three regions of interest for finite and restructuring constructions in Spanish-English bilinguals and functionally monolingual Spanish speakers**.

In what follows we report the results for finite constructions first, followed by those for restructuring constructions. We will report the most relevant results. A complete table with all the results with F1, F2, MSE values and *post hoc* test statistics is provided in Appendix B. Results for both constructions are given in **Figure 2**.

##### Finite constructions

There was a main effect of correctness approaching significance at the first ROI, and significant at the second and third ROIs [first ROI: *F*1(1,37) = 3.489; *p* = 0.07; *F*2(1,9) = 4.257; *p* = 0.046; Second ROI: *F*1(1,37) = 32.093; *p* < 0.001; *F*2(1,9) = 27.5; *p* < 0.001; Third ROI: *F*1(1,37) = 13.208; *p* < 0.001; *F*2(1,9) = 7.121; *p* = 0.001]. There were no differences by group. At the first and second ROIs, reading times for clitics in the incorrect position were longer than reading times for clitics in the correct position. At the third ROI, there were slower reading times for clitics in the correct position than in the incorrect position. For all the three ROIs no group effects or interactions by group were significant.

##### Restructuring constructions

At the first ROI, there was a main effect of correctness, with clitics in the incorrect position being read slower than clitics in the correct position [*F*1(1,37) = 287.5; *p* < 0.01; *F*2(1,39) = 318.9; *p* < 0.01]. Analyses at the second and third ROIs showed a main effect of correctness with words following a clitic in the correct position being read slower than words following an incorrect clitic [second ROI: *F*1(1,37) = 51.3; *p* < 0.01; *F*2(1,39) = 40.8; *p* < 0.001; third ROI: *F*1(1,37) = 46.7; *p* < 0.01; *F*2(1,39) = 137; *p* < 0.001]. At the third ROI there was also a significant Correctness by Group interaction in the item analysis (not significant at the subject level). Results for both constructions are represented in **Figure 2**.

### Discussion

The goal of Experiment 2 was to determine whether the performance of Spanish–English bilinguals tested in Experiment 1 differed from the performance of a group of functionally monolingual Spanish speakers tested in their L1 environment. The data show that the immersion context and the bilingual or monolingual experience of native Spanish speakers did not appear to affect the pattern of results in any significant respect. It is important to note that the Spanish–English bilinguals in Experiment 1 had been immersed in an English environment for a relatively long period, on average for 4 years. The failure to observe a difference in clitic processing for the two groups is therefore unlikely to be due to lack of an opportunity on the part of the immersed Spanish speakers to change if that change was going to occur. In addition to being a structure that is unique to the L2 Spanish, clitics appear to be a stable structure in native speakers that is not easily affected by the conditions of language use. These results are important in that they demonstrate that the clitic construction is apparently not open to the influences of the frequency of use in the way that other lexical information and parsing preferences may change in response to L2 usage (e.g., Dussias, 2003; Malt and Sloman, 2003; Dussias and Sagarra, 2007; Schmid, 2010).

## General Discussion

The goal of this study was to determine whether proficient but late English–Spanish bilinguals could process complex morphosyntactic information on-line in Spanish to the same extent as native Spanish speakers. Spanish clitic pronouns were analyzed as an example of an L2 specific structure. Previous studies have shown that proficient late bilinguals are sensitive to violations of number and grammatical gender marked in clitics (e.g., Rossi et al., 2014), but those studies were not designed to test sensitivity to the clitics’ word order. Critical to the goal of the present research, clitics vary in sentential position across different constructions. As we have argued, they represent an ideal structure to investigate whether late high-proficiency bilinguals are able to process complex morphosyntactic information in L2 that is not present in their L1. Experiment 1 demonstrated that when late English–Spanish bilinguals read sentences in Spanish, they were sensitive to violations of clitic position, like native speakers. These results indicate that late bilinguals can access and utilize L2-specific information to detect ungrammaticalities while processing linguistic information on-line, even for grammatical structures that are unique to the L2. The results also converge with past research on clitic production which shows that L2 speakers rarely make placement errors (For L2 learners: Grüter, 2006; Grüter and Crago, 2012; Grüter et al., 2012; for L1 acquisition Varlokosta et al., 2016; for agrammatic speakers Rossi, 2013). Recent behavioral and ERP studies are in line with the present findings in showing that a subset of highly proficient, but late, L2 speakers of languages that encode clitic pronouns was sensitive to grammatical gender and number agreement marked on clitic pronouns (Coughlin and Tremblay, 2013; Rossi et al., 2014). Critically, in line with previous off-line behavioral studies (e.g., Duffield and White, 1999; Duffield et al., 2002; Montrul, 2010), the results of Experiment 1 demonstrated that proficient late bilinguals are able to process word order for grammatical structures that are unique to the L2. Like native speakers, they were sensitive to violations of clitic position in restructuring sentences (in which the surfacing word order is similar to the one observed for English weak pronouns), and also for finite sentences in which there is no overlap between Spanish and English.

The results of Experiment 2, with monolingual Spanish speakers living in Spain in their L1 environment, were virtually identical to the results for the immersed native Spanish speaking bilinguals living in the US in Experiment 1. For the purpose of the present study, these results are important in that they demonstrate that the clitic construction is relatively stable and apparently not open to the influences of the frequency of use in the way that lexical information or parsing preferences may change in response to L2 usage (e.g., Dussias, 2003; Malt and Sloman, 2003; Dussias and Sagarra, 2007; Schmid, 2010). As noted earlier, clitic pronouns are complex grammatical constructions which have been shown to impose processing demands during L1 acquisition (Pérez-Leroux et al., 2011), and are particularly vulnerable for agrammatic speakers (Rossi, 2013). As such, we propose that clitic pronouns may provide a more stringent context in which to test models of complete L2 acquisition. Finding that late and proficient bilinguals are able to process clitic violations fully upon their initial encounter in a sentence, suggests the complexity of the structure itself does not prevent native-like performance.

Other behavioral and neurophysiological studies also demonstrate that L2 speakers can largely process L2-unique structures, such as grammatical gender, in a native-like manner (e.g., Tokowicz and MacWhinney, 2005; Tolentino and Tokowicz, 2011; Foucart and Frenck-Mestre, 2012). Foucart and Frenck-Mestre (2012) reported a series of ERP and eye-tracking experiments in which they tested the ability of English–French bilinguals to process grammatical gender on line in local and non-local contexts. Because English is not marked for gender, this is a structure that is unique to French for these bilinguals. Results showed that English–French bilinguals showed a P600 for gender agreement in local contexts. Foucart and Frenck-Mestre concluded that L2 speakers can access and process grammatical structures as native speakers, even if those structures are not present in their L1. Similar results on the processing of grammatical gender marked on clitic pronouns were reported by Rossi et al. (2014). Taken together, the results of the two experiments we report here demonstrate that highly proficient L2 speakers are able to access grammatical structures on-line during sentence processing, even when the structure is unique to the L2 and even when the L2 imposes language-specific word order constraints.

In this study we employed on-line behavioral measures as a first step to unveil sensitivity to clitic pronouns word-order violation. Results show overall that late L2 learners are sensitive to this aspect of the L2 syntax. However, behavioral measures provide relatively little information about the nature of syntactic processing. More time-sensitive neural methodologies such as EEGs (analyzed both in the time ERPs- and in the frequency domain), or functional magnetic resonance imaging -fMRI- that capture syntactic processing with high temporal and spatial precision, might be more suitable to reveal mechanisms related to syntactic processing that might be otherwise not be detectable with behavioral methodologies. Recent work in our lab (Rossi and Prystauka, unpublished), also analyzed the oscillatory signal related to clitic pronoun processing in native Spanish speakers and late English L2 learners of Spanish as a new methodology that allows an even more refined analysis of syntactic processing in variable populations such as L2 speakers. The results we report and those of the studies that we have reviewed suggest that it is possible for late bilinguals to become sensitive to subtle aspects of the L2 grammar. At the same time, late bilinguals, including those tested in Experiment 1, were not identical to native speakers, showing lower accuracy rates. Understanding the differences that remain between native and L2 processing is a topic that will require additional research.

A framework for investigating differences between L1 and L2 can be found in the recent psycholinguistic literature that examines prediction processes. Results from a variety of studies on native speaker performance converge on the idea that sentence processing is a dynamic process during which speakers are sensitive to linguistic cues available in the input, integrating this information to formulate predictions that guide comprehension (e.g., MacDonald et al., 1994; Trueswell and Tanenhaus, 1994; Altmann and Kamide, 1999). A number of studies have shown that the ability to access morphosyntactic information and use it predictively is a central feature of native language processing (Wicha et al., 2004; Lew-Williams and Fernald, 2007). On this perspective, the question of whether late bilinguals are able to utilize linguistic cues predictively has been the focus of recent investigations examining L2 prediction abilities (Kaan et al., 2010; Martin et al., 2013; Hopp, 2014). For example, Dussias and Cramer Scaltz (2008) showed that L2 speakers are able to exploit verb-subcategorization information to predict upcoming information. Likewise, Hopp (2014) showed that a subset of L2 German speakers was able to exploit gender cues marked on the determiner to correctly anticipate looks to a target picture, suggesting that it is possible for L2 speakers to use grammatical information predictively. The present experiments were not designed to test alternative claims about prediction in sentence processing. However, as noted earlier, the data in Experiment 1 suggest that only native Spanish speakers show longer reading times at the second ROI in finite constructions when clitic position was previously violated. L2 speakers of Spanish do not show this effect, suggesting that a critical difference between late bilinguals and native speakers may be in the ability to maintain temporarily active alternative interpretations.

## Conclusion

We have shown that late bilinguals are able to process a subtle and stable aspect of the L2 grammar. In addition, the data suggest that the questions that have been asked about constraints on late L2 acquisition may be inadequate in capturing the full complexity of language processing. Whether there are constraints on L2 processing may depend on the nature of the structure tested and the methodology chosen to address specific questions. The recent literature demonstrates remarkable plasticity in the way that bilinguals process grammar, not only in the L2, but also in the L1, suggesting that some structures are more open to cross-language influences than other structures. Few of the studies that have investigated these issues in L2 speakers have examined performance in the L1. Those that have, suggest that there are changes to native language processing that may reveal the basis of the observed plasticity (e.g., Dussias and Sagarra, 2007; Kroll et al., 2015b). A goal in future research will be to identify those aspects of the grammar that are likely to reflect these changes and those that may be constrained by the history of a bilingual’s language experience.

## Author Contributions

ER: ER prepared the study design, she collected and analyzed the data. ER wrote the manuscript. MD: MD took part in contributing to the manuscript writing and to its revision. JK: JK contributed to the study design, the manuscript writing and revision. PD: PD contributed to the study design, the manuscript writing and revision.

## Conflict of Interest Statement

The authors declare that the research was conducted in the absence of any commercial or financial relationships that could be construed as a potential conflict of interest.
