# Supplementary material for: Late Bilinguals Are Sensitive to Unique Aspects of Second Language Processing: Evidence from Clitic Pronouns Word-Order
Source: Front Psychol. 2017 Mar 17;8:342. doi: 10.3389/fpsyg.2017.00342 (PMC5355469; doi:10.3389/fpsyg.2017.00342)
Supplement: Supplementary file 1 [file Data_Sheet_1.docx]

**Appendix A**

Experiment 1: F1 and F2 statistics for self-paced RTs for both constructions.

|  |  | ***FINITE CONSTRUCTIONS*** | | | | | ***RESTRUCTURING CONSTRUCTIONS*** | | | |
| --- | --- | --- | --- | --- | --- | --- | --- | --- | --- | --- |
| ***F1 statistics*** | | | | | | | | | | |
| **FIRST ROI** | **df** | **Mean Square** | | **F1** | | **Sig.** | **Mean Square** | | **F1** | **Sig.** |
| Correctness | 1 | 13024.423 | | 11.654 | | **0.002** | 2670461.435 | | 91.115 | **p<0.01** |
| Correctness* Group | 1 | 7988.885 | | 7.148 | | **0.011** | 9184.666 | | 0.313 | 0.579 |
| Error(Correctness) | 37 | 1117.609 | |  | |  | 29308.629 | |  |  |
| ^@^L1*Correctness | 19 |  | | t=-0.13 | | 0.474 |  | |  |  |
| ^@^L2*Correctness | 20 |  | | t=-3.59 | | **0.002** |  | |  |  |
| **SECOND ROI** | **df** | **Mean Square** | | **F1** | | **Sig.** | **Mean Square** | | **F1** | **Sig.** |
| Correctness | 1 | 14254.657 | | 2.007 | | 0.165 | 39055.282 | | 22.862 | **p<0.01** |
| Correctness* Group | 1 | 51276.323 | | 7.22 | | **0.011** | 6273.756 | | 3.672 | 0.063 |
| Error(Correctness) | 37 | 7101.652 | |  | |  | 1708.335 | |  |  |
| ^@^L1*Correctness | 19 |  | | t=-3.88 | | **0.011** |  | |  |  |
| ^@^L2*Correctness | 20 |  | | t=-0.760 | | 0.456 |  | |  |  |
| **THIRD ROI** | **df** | **Mean Square** | | **F1** | | **Sig.** | **Mean Square** | | **F1** | **Sig.** |
| Correctness | 1 | 18945.608 | | 8.795 | | **0.005** | 77572.552 | | 36.954 | **p<0.01** |
| Correctness* Group | 1 | 583.018 | | 0.271 | | 0.606 | 3102.514 | | 1.478 | 0.232 |
| Error(Correctness) | 37 | 2154.151 | |  | |  | 2099.147 | |  |  |
|  | | | ***FINITE CONSTRUCTIONS*** | | | | | ***RESTRUCTURING CONSTRUCTIONS*** | | |
| ***F2 statistics*** | | | | | | | | | | |
| **FIRST ROI** | **df** | **Mean Square** | | **F2** | **Sig.** | | **Mean Square** | | **F2** | **Sig.** |
| Correctness | 1 | 62331.025 | | 13.371 | **0.001** | | 912946.225 | | 69.509 | **p<0.01** |
| Error(Correctness) | 39 | 4661.656 | |  |  | | 13134.254 | |  |  |
| Group | 1 | 26496.756 | | 13.422 | **0.001** | | 4182208.9 | | 167.737 | **p<0.01** |
| Error(Group) | 39 | 1974.176 | |  |  | | 24933.198 | |  |  |
| Correctness* Group | 1 | 7770.156 | | 2.518 | 0.121 | | 139417.056 | | 5.324 | **0.026** |
| Error(Correctness*Group) | 39 | 3085.256 | |  |  | | 26185.656 | |  |  |
| **SECOND ROI** | **df** | **Mean Square** | | **F2** | **Sig.** | | **Mean Square** | | **F2** | **Sig.** |
| Correctness | 1 | 33828.764 | | 4.142 | **0.049** | | 25603.6 | | 15 | **p<0.01** |
| Error(Correctness) | 39 | 8167.815 | |  |  | | 1706.911 | |  |  |
| Group | 1 | 4834.502 | | 0.931 | 0.34 | | 60918.025 | | 16.993 | **p<0.01** |
| Error(Group) | 39 | 5190.136 | |  |  | | 3584.99 | |  |  |
| Correctness* Group | 1 | 85956.077 | | 20.213 | **p<0.01** | | 32007.306 | | 17.878 | **p<0.01** |
| Error(Correctness*Group) | 39 | 4252.474 | |  |  | | 1790.303 | |  |  |
| **THIRD ROI** | **df** | **Mean Square** | | **F2** | **Sig.** | | **Mean Square** | | **F2** | **Sig.** |
| Correctness | 1 | 21091.056 | | 3.513 | 0.068^a^ | | 36300.625 | | 7.964 | **p<0.01** |
| Error(Correctness) | 39 | 6004.38 | |  |  | | 4558.061 | |  |  |
| Group | 1 | 32205.625 | | 12.519 | **0.001** | | 353158.056 | | 22.272 | **p<0.01** |
| Error(Group) | 39 | 2572.455 | |  |  | | 15856.518 | |  |  |
| Correctness* Group | 1 | 722.5 | | 0.188 | 0.667 | | 950.625 | | 0.159 | 0.692 |
| Error(Correctness*Group) | 39 | 3838.138 | |  |  | | 5983.112 | |  |  |

LEGEND: ^@^ Post-hoc pairwise comparisons; ^a^ trending significance

**Appendix B**

Experiment 2: F1 and F2 statistics for both constructions.

|  |  | ***FINITE CONSTRUCTIONS*** | | | ***RESTRUCTURING CONSTRUCTIONS*** | | |
| --- | --- | --- | --- | --- | --- | --- | --- |
| ***F1 statistics*** | | | | | | | |
| **FIRST ROI** | **df** | **Mean Square** | **F1** | **Sig.** | **Mean Square** | **F1** | **Sig.** |
| Correctness | 1 | 3585.89 | 3.489 | 0.07^a^ | 3299164.352 | 287.534 | **p<0.01** |
| Correctness* Group | 1 | 1234.698 | 1.201 | 0.28 | 7459.583 | 0.65 | 0.425 |
| Error(Correctness) | 37 | 1027.656 |  |  | 11474.007 |  |  |
| **SECOND ROI** | **df** | **Mean Square** | **F1** | **Sig.** | **Mean Square** | **F1** | **Sig.** |
| Correctness | 1 | 161315.005 | 32.093 | **p<0.01** | 61533.237 | 51.338 | **p<0.01** |
| Correctness* Group | 1 | 3114.172 | 0.62 | 0.436 | 827.839 | 0.691 | 0.411 |
| Error(Correctness) | 37 | 5026.466 |  |  | 1198.592 |  |  |
| **THIRD ROI** | **df** | **Mean Square** | **F1** | **Sig.** | **Mean Square** | **F1** | **Sig.** |
| Correctness | 1 | 13754.948 | 13.208 | **0.001** | 84001.701 | 46.705 | **p<0.01** |
| Correctness* Group | 1 | 14.32 | 0.014 | 0.907 | 1970.316 | 1.095 | 0.302 |
| Error(Correctness) | 37 | 1041.389 |  |  | 1798.56 |  |  |
|  |  | ***FINITE CONSTRUCTIONS*** | | | ***RESTRUCTURING CONSTRUCTIONS*** | | |
| ***F2 statistics*** | | | | | | | |
| **FIRST ROI** | **df** | **Mean Square** | **F2** | **Sig.** | **Mean Square** | **F2** | **Sig.** |
| Correctness | 1 | 17598.025 | 4.257 | **0.046** | 6726050.156 | 318.926 | **p<0.01** |
| Error(Correctness) | 39 | 4133.708 |  |  | 21089.704 |  |  |
| Group | 1 | 507.656 | 0.658 | 0.422 | 36000 | 3.454 | 0.071^a^ |
| Error(Group) | 39 | 771.576 |  |  | 10422.048 |  |  |
| Correctness* Group | 1 | 832.656 | 0.201 | 0.656 | 30858.025 | 1.843 | 0.182 |
| Error(Violation *Group) | 39 | 4140.704 |  |  | 16743.772 |  |  |
| **SECOND ROI** | **df** | **Mean Square** | **F2** | **Sig.** | **Mean Square** | **F2** | **Sig.** |
| Correctness | 1 | 219040 | 27.5 | **p<0.01** | 150031.877 | 40.808 | **p<0.01** |
| Error(Correctness) | 39 | 7965.179 |  |  | 3676.54 |  |  |
| Group | 1 | 7209.225 | 3.092 | 0.087 | 9294.877 | 12.167 | **p<0.01** |
| Error(Group) | 39 | 2331.231 |  |  | 763.963 |  |  |
| Correctness* Group | 1 | 82.656 | 0.026 | 0.873 | 1473.189 | 0.743 | 0.394 |
| Error(Correctness*Group) | 39 | 3212.682 |  |  | 1982.474 |  |  |
| **THIRD ROI** | **df** | **Mean Square** | **F2** | **Sig.** | **Mean Square** | **F2** | **Sig.** |
| Correctness | 1 | 25363.814 | 7.121 | **0.011** | 1045633.064 | 137.551 | **p<0.01** |
| Error(Correctness) | 39 | 3561.721 |  |  | 7601.808 |  |  |
| Group | 1 | 20782.202 | 14.41 | **0.001** | 135693.377 | 23.229 | **p<0.01** |
| Error(Group) | 39 | 1442.25 |  |  | 5841.53 |  |  |
| Correctness* Group | 1 | 165.039 | 0.045 | 0.833 | 222345.377 | 20.294 | **p<0.01** |
| Error(Correctness*Group) | 39 | 3651.767 |  |  | 10956.383 |  |  |

LEGEND: ^@^ Post-hoc pairwise comparisons; ^a^ trending significance
